# Upregulation of BMSCs Osteogenesis by Positively-Charged Tertiary Amines on Polymeric Implants *via* Charge/iNOS Signaling Pathway

**DOI:** 10.1038/srep09369

**Published:** 2015-03-20

**Authors:** Wei Zhang, Na Liu, Haigang Shi, Jun Liu, Lianxin Shi, Bo Zhang, Huaiyu Wang, Junhui Ji, Paul K. Chu

**Affiliations:** 1 Technical Institute of Physics and Chemistry, Chinese Academy of Sciences, Beijing 100190, China; 2 Stomatology Department of the General Hospital of Chinese PLA, 28 FuXing Road, Beijing 100853, China; 3 Department of Physics & Materials Science, City University of Hong Kong, Tat Chee Avenue, Kowloon, Hong Kong, China

## Abstract

Positively-charged surfaces on implants have a similar potential to upregulate osteogenesis of bone marrow-derived mesenchymal stem cells (BMSCs) as electromagnetic therapy approved for bone regeneration. Generally, their osteogenesis functions are generally considered to stem from the charge-induced adhesion of extracellular matrix (ECM) proteins without exploring the underlying surface charge/cell signaling molecule pathways. Herein, a positively-charged surface with controllable tertiary amines is produced on a polymer implant by plasma surface modification. In addition to inhibiting the TNF-α expression, the positively-charged surface with tertiary amines exhibits excellent cytocompatibility as well as remarkably upregulated osteogenesis-related gene/protein expressions and calcification of the contacted BMSCs. Stimulated by the charged surface, these BMSCs display high iNOS expressions among the three NOS isoforms. Meanwhile, downregulation of the iNOS by L-Can or siRNA inhibit osteogenic differentiation in the BMSCs. These findings suggest that a positively-charged surface with tertiary amines induces osteogenesis of BMSCs *via* the surface charge/iNOS signaling pathway in addition to elevated ECM protein adhesion. Therefore, creating a positively-charged surface with tertiary amines is a promising approach to promote osseointegration with bone tissues.

As an excellent cell source for bone regeneration and repair^1^,^2^, bone marrow-derived mesenchymal stem cells (BMSCs) have recently attracted large interest due to the potential to control and regulate BMSCs osteogenic differentiation on orthopedic implants^3^,^4^. An external electric field can induce the expression of the bone marker genes in BMSCs^5^,^6^ and promote bone tissue regeneration^7^,^8^,^9^. Moreover, the positively-charged materials have been shown to specifically direct BMSCs to differentiate to accomplish osteogenesis and promote bone regeneration^10^,^11^,^12^,^13^,^14^,^15^. Thus, the construction of a positively-charged surface on a polymeric implant to form a local biochemical and electrical micro-environment has a similar potential to upregulate osteogenesis of the contacted BMSCs being capable of enhancing osseointegration^10^,^11^,^12^. Most positively-charged surfaces on bone implants are derived from coatings with chitosan^11^,^12^, polyelectrolyte^13^,^14^, and polyallylamine^15^, but unfortunately, these coatings cannot satisfy the requirements in many aspects such as matching mechanical strength, biocompatibility, toughness against delamination, and release of small molecules. Recent research activities^11^,^12^,^13^,^14^,^15^ have focused on cell adhesion and osteoconduction on positively-charged surfaces and it has been shown that their osteogenesis functions^12^,^15^ are primarily attributed to the elevated adhesion of extracellular matrix (ECM) proteins such as fibronectin on the surface in the initial phase^13^,^14^,^15^. Some studies^5^,^6^, however, have revealed that a simple electrical stimulus can also induce osteogenic expressions of BMSCs in the absence of ECM protein change *in vivo*. Nevertheless the underlying mechanism is still not well understood and that governing osteogenesis regulation of BMSCs on a positively-charged surface should be further explored.

Derived from the macromolecular chains of polymeric orthopedic implants, tertiary amines generated by the surface plasma modification can circumvent the shortcomings plaguing coatings containing chitosan, polyelectrolyte, and polyallylamine. Since these tertiary amines are protonated at the biological pH of 7.5^16^,^17^, a surface with tertiary amines offers a local biochemical and electrostatic environment favorable to BMSCs. Similar to an external electrical stimulus, this local environment can stimulate and guide BMSCs to differentiate to attain osteogenesis on the implant. Nitric oxide (NO) is essential to many biological processes^18^,^19^ and the NO signaling molecule plays an important role in bone metabolism^19^,^20^,^21^ and mediates osteogenesis-related gene/protein expressions and calcification of BMSCs^18^,^22^,^23^. Recent findings^24^,^25^ show that in order to generate NO, electron transfer must happen in the nitric oxide synthase (NOS) domains from the NOS FMN sub-domain to the heme. In this study, it is assumed that electron transfer in NOS is affected by the local biochemical and electrical environment, thereby resulting in BMSCs exhibiting different NOS isoform expressions to further signal the expressions of bone osteogene markers. These assumptions are verified by creating a positively-charged surface with tertiary amines galore on a polymeric implant by surface plasma modification. The regulating effects on the osteogenic differentiation of BMSCs are systematically studied to elucidate the mechanism *via* the surface charge/NOS signaling pathway.

## Results

### Design and formation of positively-charged surfaces with tertiary amines

**Figure 1:**
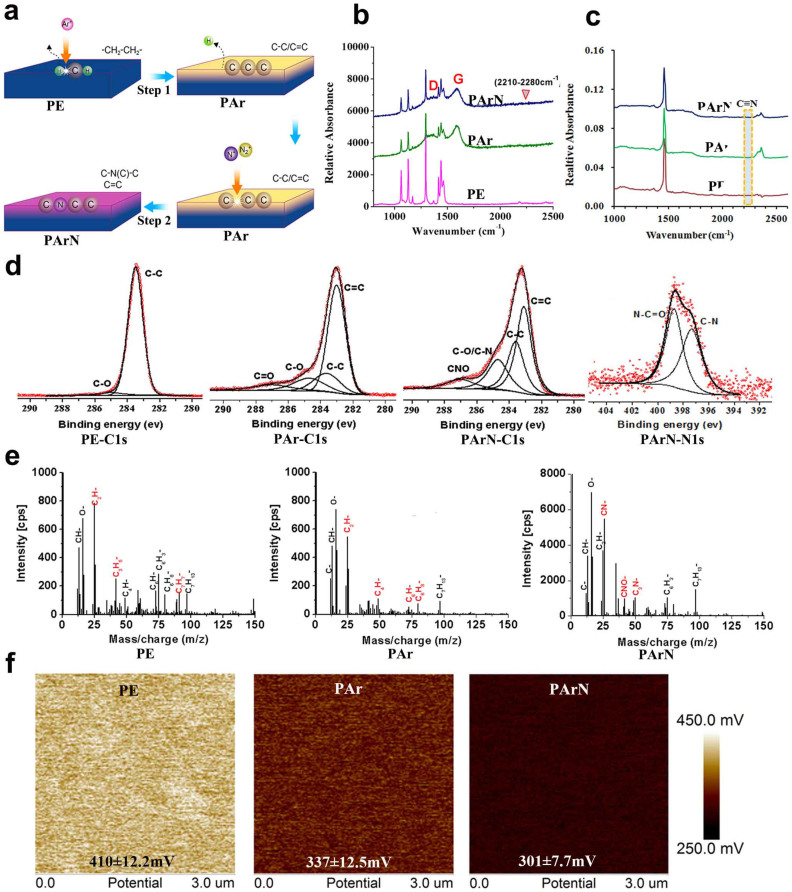
Preparation procedures and surface characteristics of the positively-charged surface with tertiary amines. (a) Schematic diagram illustrating the preparation procedure: (1) The pyrolytic carbon is formed on the PE substrate by argon plasma treatment (6.14 × 10^15^ ions/cm^2^) for 10 minutes to obtain the PAr sample and (2) The targeted nitrogen functionalities are generated by nitrogen PIII and the sample is designated as PArN. (b) Raman spectra of PE, PAr, and PArN, suggesting the formation of pyrolytic carbon structure after argon plasma treatment. (c) ATR-FTIR spectra of PE, PAr, and PArN. Some C = N and C≡N bonds are not detected at about 1650 cm^−1^ and 2230 cm^−1^. (d) Deconvoluted XPS spectra revealing that C = C, CN and CNO bonds are formed on PArN. (e) TOF-SIMS negative ion spectra of PE, PAr, and PArN, further showing CN-, CNO- and C_3_N- fragments attributable to C-N(C)-C and N-C = O, and but little C = N and C≡N. (f) KFM analysis showing the relative surface potential of PE, PAr, and PArN as follows: PE = 410 ± 12.2 mV; Par = 337 ± 12.5 mV, and PArN = 301 ± 7.7 mV.

Surface plasma modification is an excellent approach to modify the chemical structure^26^ and produce the targeted nitrogen functionality^27^,^28^,^29^. It has been demonstrated that a nitrogen plasma-modified surface can serve as a powerful artificial microenvironment to regulate osteogenic differentiation of osteoblasts^30^,^31^,^32^. However, the inherently complicated chemical structure of many biopolymers containing C-H, C-O, C = O, C-N, and N-H bonds makes it difficult to controllably construct the specific nitrogen functionality to attain the desirable biological outcome^33^,^34^. Therefore, previous results concerning the effects of plasma-generated nitrogen functionalities such as primary, secondary, and tertiary amines on bone cells are ambiguous and sometimes contradictory^30^,^31^,^32^,^33^. In this study, the inherent chemical bonds are dissociated and O and H are sputtered off by argon ion bombardment to convert the polymeric surface into a simple carbonaceous structure^28^,^35^. The preparation procedures are illustrated by Step 1 in Fig. 1a. Raman scattering reveals that the D and G bands at 1330 cm^−1^ and 1580 cm^−1^ are typical of pyrolytic carbon (Fig. 1b, PAr). The broadened D bands of PAr and PArN indicate that there are many structural defects and lack of spatial uniformity and disappearance of the 2D band also suggests a lack of spatial uniformity. The G band is often detected from sp^2^ systems originating from in-plane vibrations and suggests C = C bond formation in PAr. ATR-FTIR and XPS also confirm that a pyrolytic carbon structure is produced on the PE substrate after Ar ion bombardment (Figs. 1c and 1d, PAr). By considering the decreased peaks corresponding to vibrations of -CH_2_ at 1061 nm, 1130 nm, 1293 nm, and 1439 nm in the Raman spectra (Fig. 1b, PAr), larger amounts of C-, C_4_H- and C_6_H-, and reduced amounts of C_3_H_5_- and C_7_H_7_- ions in the TOF-SIMS spectrum (Fig. 1e, PAr), it can be inferred that during argon ion bombardment, dissociation of the chemical bonds occurs and hydrogen is sputtered off from the PE molecular chain leading to the formation of pyrolytic carbon.

The pyrolytic carbon serves as the platform to produce tertiary amines on the polymeric surface. The procedures are illustrated in Fig. 1a (Step 2). After nitrogen PIII, Raman scattering indicates that the D and G bands remain (Fig. 1b, PArN). ATR-FTIR, XPS, and TOF-SIMS performed on PArN show that the generated nitrogen functionalities mainly consist of -C-N(C)-C and N-C = O groups instead of primary and secondary amines (Figs. 1d and 1e, PArN). Tertiary amines are produced selectively on the PE in spite of the existence of N-C = O groups due to oxidation. It can be inferred that on the pyrolytic carbon structure, other nitrogen functionalities (for example, -NH_2_) can be generated by increasing the hydrogen amount in the implanted ions^27^. In addition, most of the C = C bonds remain on PArN implying that the π-conjugated structures are abundant on the PArN surface. It is possible to exploit the cation-π interaction with the protonated tertiary amines to promote the surface electrostatic potential^36^. Kelvin probe force microscopy (KFM), conducted under dry conditions, shows that the PArN surface has a larger potential than PAr and PE (Fig. 1f) and it is the main reason for the formation of tertiary amines (C-N(C)-C groups). The tertiary amine groups can be protonated in the cell culture medium at a neutral pH and displays relative high positive charge^17^. Moreover, this is an excellent indicator of the trends in cation-π interactions^36^,^37^. The promoted surface potential is partially attributed to the cation-π interactions between the π-conjugated structure and protonated tertiary amines in the cell culture medium^37^,^38^. As expected, the surface with abundant protonated tertiary amines serves as a local biochemical and electrical environment favorable to BMSCs due to the surface positive charge.

The melt behavior (Supplementary Fig. S1a) and crystal phase (Supplementary Fig. S1b) indicate that the pyrolytic carbon on PAr and PArN possess a network structure with cross-linking thus increasing the stability of the films in water (essential to biological applications). Therefore, our strategy effectively avoids alteration of the bulk properties of the implant, delamination, and release of small molecules as a result of the tertiary amines being derived from the bulk macromolecular chain. In addition, the atomic force microscopy (AFM) images (Supplementary Fig. S1c) show that the surface on PAr and PArN is rougher because Ar PIII produces cross-linked structures and during cooling, structural reorganization and contraction of the PE substrate under the pyrolytic carbon film produce interior stress between the substrate and film making the pyrolytic carbon films wrinkled.

### Apoptosis, proliferation, and inflammatory reaction of BMSCs on the positively-charged surface

**Figure 2:**
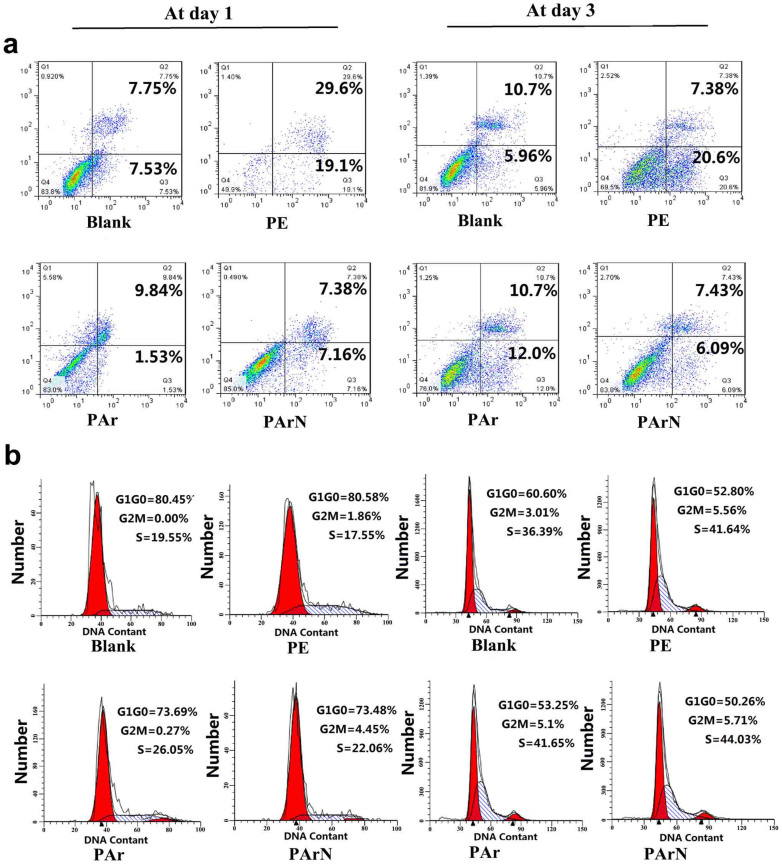
Effects of the positively-charged surface with tertiary amines on apoptosis and cell cycle of the BMSCs. (a) Cell apoptosis and (b) Cell cycle of BMSCs cultured on blank, PE, PAr, and PArN at day 1 and day 3. The figures show the cell state for triplicate experiments and 10,000 cells are analyzed.

**Figure 3:**
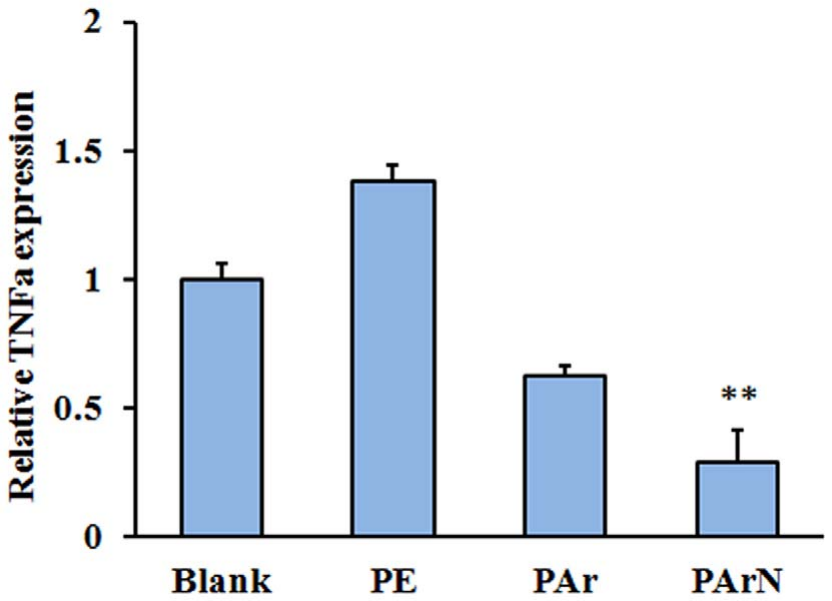
Relationship between the positively-charged surface with tertiary amines and pre-inflammatory stimuli. TNF-α expression of BMSCs on blank, PE, PAr and PArN at day 3 is measured by real time PCR, relative to the GAPDH expression and normalized to the TNF-α expression on blank, (**, p < 0.01) denote statistical significance compared to the blank group.

To determine the effects of the charged surface with tertiary amines on the biological functions of BMSCs, stem cell proliferation and osteogenic differentiation are investigated. The BMSCs are uncommitted entities capable of both self-renewal and differentiation into multiple cell lineages^1^. The local microenvironment such as surface chemical groups, surface area, and topography, and protein absorption can significantly affect the cell behavior^10^,^39^,^40^,^41^. Protein absorption is also determined by surface characteristics such as the chemical groups, topography, and surface charge^41^. In order to objectively evaluate the biological properties of the charged surface (PArN), tissue culture plastic (blank), PE, and PAr are employed as the control groups. The blank group serves as the standard control to evaluate the cellular behavior of BMSCs. PE has different morphology and chemical groups compared to PArN. Although PAr and PArN have the similar topography, the difference lies in the surface charge level stemming from the C-N(C)-C groups on the surface. Firstly, FCM is carried out to explore the effects of the charged surface on the viability, apoptosis, and proliferation of BMSCs. There is no significant difference (p > 0.05) in the proliferation and apoptosis between the blank and PArN groups demonstrating that the charged surface (PArN) with tertiary amines has excellent compatibility with BMSC cells (Fig. 2). Studies of TNF-α release related to the immunogenic response are conducted to determine the potential in biomedical and tissue engineering. PAr and PArN are found to induce low inflammatory response and TNF-α value, comparable to PE and cell culture plates (Fig. 3). It has been reported that BMSCs suppress T cell proliferation and cytokine production^42^. The other prominent factor in BMSCs-mediated immune suppression is NO^43^ which plays a key role in the macrophage functions and affects TCR signalling, cytokine receptor expression, and phenotype of T cells^44^. Our results show that the charged surface (PArN) with tertiary amines may have some capability in suppressing BMSCs inflammation by activated NO signaling.

### Positively-charged surface promoting osteogenic potential of BMSCs

**Figure 4:**
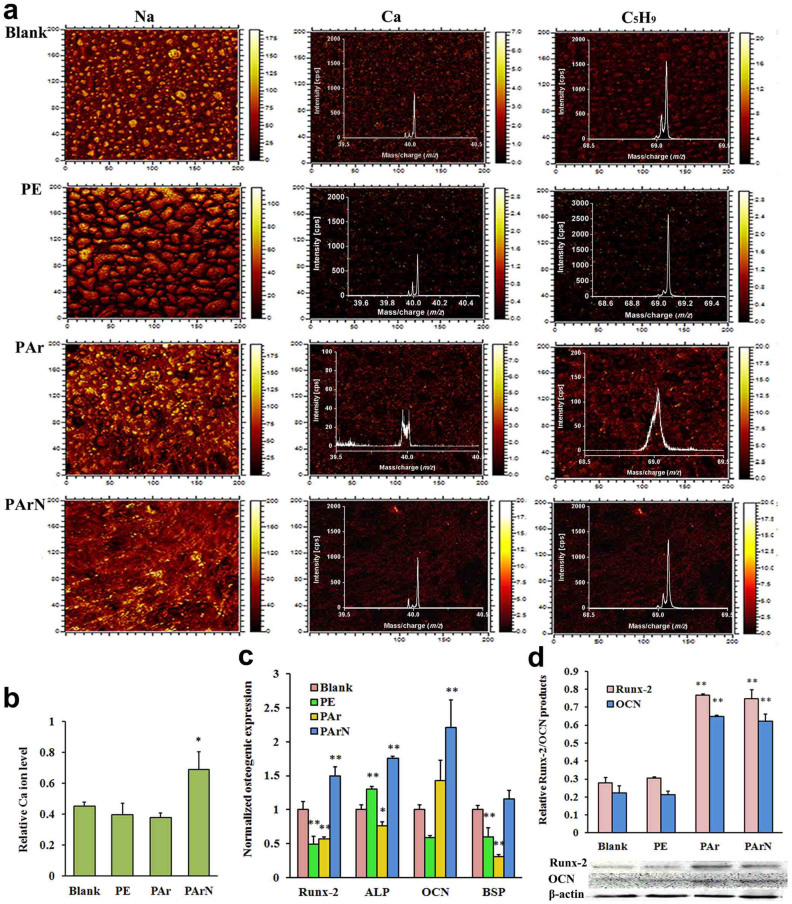
Influence of the positively-charged surface with tertiary amines on calcification and bone-related genes/protein expressions of BMSCs. (a) TOF-SIMS images of BMSs cultured for 7 days. The spectrum was the high-resolution TOF-SIMS spectra. (b) Ca ion levels normalized to the C_5_H_9_ fragment showing that there is higher Ca level on PArN than PAr and blank. The field of view is 200 μm × 200 μm. The figures show the TOF-SIMS images representative of three repeated analyses. (c) Expression levels of osteogenic markers (Runx-2, ALP, OCN, and BSP) of BMSCs at day 3 by real time PCR relative to GAPDH expression and normalized to the expressions by cells cultured on blank, showing PArN promotes osteogenic expression of BMSCs. (d) Runx-2 and OCN protein products of the cells cultured at day 7 by western blot analysis relative to β-actin, revealing PArN increases osteogenic protein products of BMSCs. (*, p < 0.05) and (**, p < 0.01) denote statistical significance compared to blank group.

In early studies involving the use ALP or Alizarin red staining to detect the osteogenic differentiation ability of stem cells, 14 to 21 days are needed and the results are not too accurate^45^. TOF-SIMS, a surface-sensitive mass spectrometric technique^46^,^47^, is conducted to monitor calcification of BMSCs cultured on the blank, PE, PAr, and PArN in the early stage (Fig. 4a). Localization of sodium can be used as a general indicator of the location of cytosol in a cell. The TOF-SIMS images show that the BMSCs have a spread morphology on PAr and PArN and round morphology on PE, suggesting that argon PIII followed by nitrogen PIII enhances BMSCs growth on the surface (Fig. 4a). The amount of calcium (*m/z* = 40) is a specific indicator of calcification. The calcium ion images show that more hydroxyapatite emerges from the blank, PAr, and PArN. The C_5_H_9_ fragment (*m/z* = 69), which is most likely an acyl chain fragment from the membrane phospholipids^46^, is homogeneous in individual cells and therefore, its density and distribution present the number and total phospholipid distribution of BMSCs on the surface. The calcium ion level normalized to C_5_H_9_ reflects the calcification ability of cells without interference of the cell number. As calculated from the high-resolution spectrum of calcium and C_5_H_9_ (Fig. 4a), BMSCs have a larger mineral concentration on PArN with tertiary amines than the blank and PE, whereas PAr with C-C and C = C groups does not possess the similar capability as PArN irrespective of biocompatibility (Fig. 4b). It is evident that the charged surface with tertiary amines (PArN) enhances cell calcification.

Observation of the mRNA expression levels of the osteogenic markers helps to distinguish the stages of differentiation and maturation of BMSCs into osteoblasts. Previous studies^48^,^49^ have shown that ALP is secreted during the early stage of osteogenic differentiation, whereas OCN, a marker of a mature osteoblast differentiation, is a necessary factor for bone calcification and mineralization. BSP is one of the major non-collagenous glycosylated phosphoproteins of the extracellular matrix in bone. It is a mineralized tissue-specific protein that is expressed in differentiated osteoblasts and appears to function in the initial mineralization of bone. In this study, expression of bone-related genes, including Runx-2, ALP, OCN and BSP, is observed to be upregulated in the PArN-BMSCs cultures after 3 days but in the PAr-BMSCs, all the gene expressions are not significantly changed compared to the blank (Fig. 4c). By monitoring the Runx-2 and OCN protein levels, the BMSCs are collected from PE, Par, and PArN at day 7. It is the matrix maturation stage of osteogenic differentiation and the ECM and stem cell functions are kept in balance. Our results (Fig. 4d) confirm that the charged surface (PArN) with tertiary amines increase bone-related protein products. By considering cell calcification, gene expression, and protein production, the positively-charged tertiary amines on PArN have significant effects on signaling BMSCs to differentiate *via* the osteogenic pathway, while the C-C and C = C groups on PAr are less effective in osteogenesis despite excellent cell attachment and proliferation. It is consistent with chitosan^12^,^50^ and polypeptide^51^ with amino groups.

## Discussion

As biomechanics and biological safety must be considered, surface modification is a viable approach, especially surface plasma modification^30^,^31^,^34^. Various plasma techniques have been employed to modify the surface chemistry to direct BMSCs proliferation and differentiation as well as to produce and organize the extracellular matrix^28^,^30^,^31^. Although nitrogen-containing groups can be generated by conventional plasma surface modification^30^,^33^, the chemical groups produced are complex and the effects on cytocompatibility and signaling induction are often inconclusive and sometimes contradictory^30^,^31^,^32^,^33^. As discussed previously, the positively-charged surface with tertiary amines can promote biocompatibility (Figs. 2 and 3) of the polymer and induce osteogenic differentiation (Fig. 4) of BMSCs. Meanwhile, other nitrogen functionalities such as -NH_2_ can be generated on the pyrolytic carbon by increasing the hydrogen amount in the implanted ions. It paves the way for better understanding the effects of the positively-charged surface on the biocompatibility and osteogenesis regulation of BMSCs.

**Figure 5:**
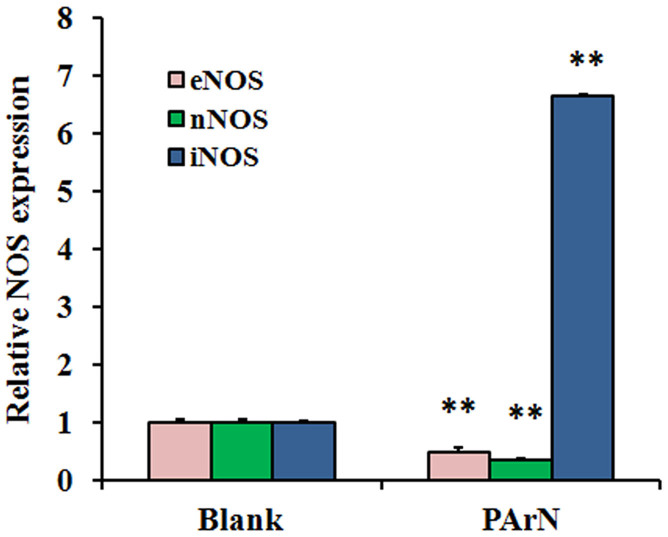
Influence of the positively-charged surface with tertiary amines on three NOS isoforms expressions in BMSCs. Expressions of eNOS/nNOS/iNOS genes of BMSCs cultured on blank and PArN are measured by real time PCR relative to GAPDH expression and normalized to the expressions on Blank. The affected NOS genes exhibit a high iNOS expression level in the local biochemical and electric stimuli environment (PArN). (**) denote the statistical significance (p < 0.01) compared to blank, respectively.

It has been reported that BMSCs osteogenic upregulation is attributed to the elevated ECM protein adhesion on a positively-charged surface^13^,^14^,^15^ but evidently, this cannot totally explain the fact that an external electric field induces the osteogenic expression of BMSCs without a supporter for ECM proteins adhesion *in vivo*^5^,^6^. Since the NO signaling molecule is a significant regulator in bone metabolism^19^,^20^,^21^, it is believed that NOS plays a pivotal physiological role in osteogenesis-related gene/protein expressions (Figs. 4c and 4d) and calcification (Figs. 4a and 4b) of BMSCs. Recent studies^24^,^25^ demonstrate that NOS are modular enzymes containing attached flavoprotein and heme (NOSoxy) domains. To generate NO, the NOS FMN sub-domain must interact with the NOSoxy domain to deliver electrons to the heme^24^,^25^. The electron transfer among NOS domains must be affected when the BMSCs are located in the biochemical and electrical environment. The affected NOS genes, in turn, exhibit variant NOS isoforms expression and NO product. Figure 5 clearly shows that the biochemical and electrical stimulus signals BMSCs to display different NOS expression levels in the three isoforms (eNOS, nNOS and iNOS)^18^,^19^. That is, there is a higher level of iNOS expression but lower levels of eNOS and nNOS on PArN than those on the blank. This is partially affirmed by the higher level of NO secretion under the electromagnetic field than the control and the activated NO signaling in osteoblasts exposed to the electric field^52^. Therefore, there is evidence that surface charges mediate the NOS expression in BMSCs.

**Figure 6:**
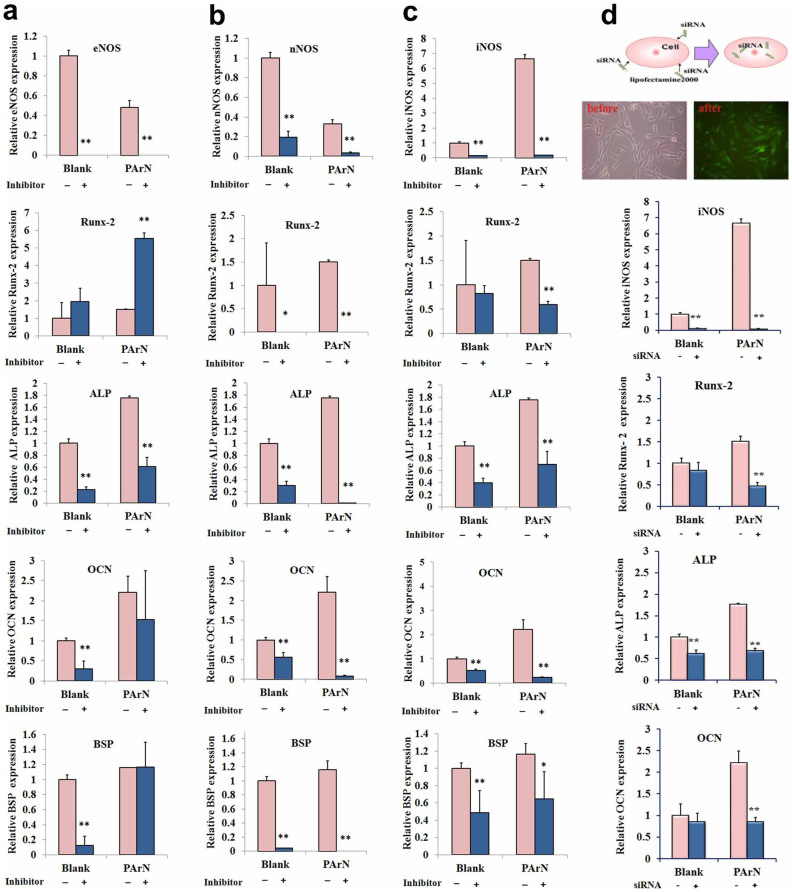
Osteogenic differentiation of BMSCs on the positively-charged surface with tertiary amines in the presence of the NOS inhibitor or siRNA-iNOS transfection. Expressions of osteogenic markers (Runx-2, ALP, OCN, and BSP) of BMSCs cultured in the presence and absence of the NOS inhibitor or siRNA-iNOS transfection in the cell culture medium for 3 days are measured by real time PCR relative to GAPDH expression and normalized to the expressions on Blank without the NOS inhibitor and siRNA. (a) eNOS inhibitor (L-NAME). (b) nNOS inhibitor (Sper). (c) iNOS inhibitor (L-Can). (d) siRNA-iNOS transfection. The bottom left illustrates transfection of siRNA-iNOS with GFP gene into BMSCs and microscopic images of the cells before and after siRNA transfection. (*) and (**) denote two statistical significance (p < 0.05 and p < 0.01), respectively, compared to the sample without NOS inhibitors and siRNA.

All three NOS isoforms are expressed in bone and the role of NO in bone metabolism has been confirmed in intact animals with single-gene deletion of NOS isoforms^53^. However, which subtype of the affected NOS helps to regulate pro-inflammation (Fig. 3) and bone formation (Fig. 4) of BMSCs stimulated by the positively-charged surface (PArN) is unknown. Herein, the effects of the three isoforms on osteo-genetic expression in the presence and absence of NOS inhibition is studied in order to disclose the signaling pathway of NO. Recent studies^54^ indicate that targeted deletion of the eNOS isoform in mice leads to an osteoblast-driven mild osteoporotic bone phenotype. As shown in Fig. 6a, the same tendency is observed from the blank and PArN groups. Stem cells exposed to L-NAME increase the mRNA expression of Runx-2 but significantly reduce the ALP expression. Therefore, inhibition of eNOS has little effects on the OCN and BSP expressions in BMSCs cultured on PArN. When the nNOS inhibitor (Sper) is added to the cell culture medium instead of L-NAME, the nNOS expression is also suppressed on both the blank and PArN (Fig. 6b). These results are partially evidenced by those obtained from mice with global deletion of nNOS^55^, showing increased bone mass due to a reduced bone turnover rate and a phenotype that is repeated in mice with deletion of all three NOS genes. The same change observed from the blank and PArN indicates that nNOS is not the signal pathway for the positively-charged surface with tertiary amines (PArN) concerning regulation of the BMSCs osteogenic expression.

**Figure 7:**
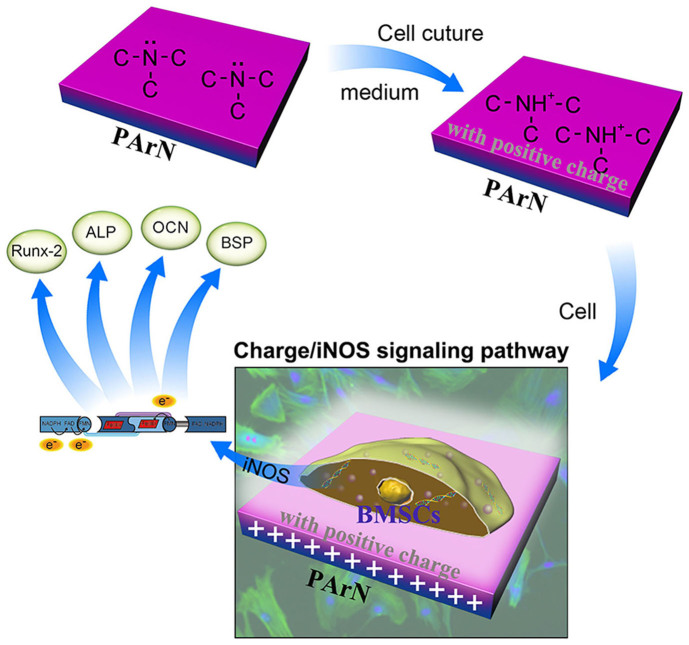
Mechanical illustration of the positively-charged surface with tertiary amines upregulating osteogenic differentiation of BMSCs *via* the iNOS pathway signaled by the surface charge.

Studies^56^ in iNOS knockout mice illustrate that iNOS-derived NO activates osteoclasts in inflammatory bone disease and plays a role in the catabolic response of bones to lower the estrogen levels. iNOS derived NO also stimulates fracture healing and recovery of bone mass after unloading-induced bone loss. Therefore, the molecular mechanism pertaining to the osteogenic capacity enhancement is studied in relationship to the increase in iNOS in PArN. iNOS and NO regulate osteogenesis during differentiation of osteoblasts. In the differentiation stage, osteoblasts can express iNOS mRNA and release NO. Figure 6c shows inhibition of iNOS and reduction of the mRNA expression levels of Runx-2, OCN, and BSP in BMSCs cultured on both the blank and PArN groups, although expression levels (Supplementary, Figure S2) of eNOS and nNOS are increased at this time. Whether genetic silencing of the iNOS expression using siRNA can reproduce the effects of iNOS inhibitor is probed. As expected, transfection of BMSCs with the 200 nM siRNA mixture can generate a knockdown of iNOS mRNA. Osteogenic differentiation of BMSCs is significantly attenuated after siRNA-iNOS transfection (Fig. 6d) having similar effects with L-Can (Fig. 6c). According to our analysis (Figs. 6c and 6d), treatment by L-Can or knockdown of iNOS by siRNA can suppress osteogenesis of BMSCs stimulated by the charged surface with tertiary amines (PArN), while PArN can activate iNOS thereby promoting bone formation and related genes such as Runx-2, ALP, OCN. The data indicate that iNOS is important to mouse fracture healing^56^ and reflect that activation of iNOS may be attributed to the biochemical and electrical stimuli arising from the tertiary amines which are sufficient to increase the osteoblast transition of BMSCs. Therefore, exogenic siRNA blocks regulation of the positively-charged surface with tertiary amines on osteogenic differentiation of BMSCs confirming that iNOS is a key signal pathway to induce BMSCs to express osteogenesis. In summary, a positively-charged surface with tertiary amines created on a polymeric orthopedic implant shows the immense potential as a local biochemical and electrical environment to activate iNOS expression and signal BMSCs to differentiate *via* the osteogenic pathway, as illustrated in Fig. 7. Therefore, creating a positively-charged surface with tertiary amines is a promising approach to promote osseointegration with bone tissues.

## Methods

### Ethics statement

Male Sprague-Dawley (SD) rats, weighing 200–300 g, were used in the present study. The animals were kept at 25°C in a 12 h light-dark cycle. They had free access to food and water. All the experiments were performed following the policy approved by the National Institutes of Health Intramural Animal Use and Care Committee (IACUC-2012-047). All experimental procedures involving animals and their care were carried out in accordance with the Guide for the Care and Use of Laboratory Animals of the National Institutes of Health.

### Preparation of the charged surface with tertiary amine

The polyethylene (PE) pellets as a model of the polymeric implant were purchased from Lucoil Chemical (Grade 277–73), press molded into 10 cm × 10 cm pieces with a thickness of 0.5 mm, and ion implanted using a Kauffman ion source. The base vacuum was less than 1.0 × 10^−5^ Pa. High-purity argon (99.99%) was introduced into the ion source and argon plasma immersion ion implantation (PIII) with an ion implant fluence of 6.14 × 10^15^ ions/cm^2^ was conducted at 5 kV for 10 min. Calibration of the ion implant fluence was accomplished by a Faraday cup and the working pressure in the vacuum chamber was 2.0 × 10^−2^ Pa during ion implantation. Afterwards, high-purity nitrogen (99.99%) was introduced in place of argon, and nitrogen PIII was conducted with the same ion implant fluence. Afterwards, the samples remained in vacuum for 10 min. The argon ion-implanted PE was denoted as PAr whereas the PE implanted with both argon and nitrogen was labeled as PArN.

### Surface physicochemical Characterization

The Raman spectra were acquired on an inVia-Reflex (Renishaw) at an excitation wavelength of 532 nm and ATR-FTIR (attenuated total-reflection Fourier transform infrared) spectra were obtained on an Excalibur 3100 (Varian). The elemental chemical composition and chemical structure were determined by X-ray photoelectron spectroscopy (XPS, PHI QUANTERA-II equipped with a monochromatic Al K_α_ source). The analyzer was operated at a pass energy (Ep) of 280 eV for wide scans and 26 eV for fine scans leading to an instrumental resolution of 1.00 eV for wide scans and 0.025 eV for fine scans. The data were collected at a take-off angle of 45° and data analysis and multi-peak fitting were performed by the Multipak software. Time-of-flight secondary ion mass spectrometry (TOF-SIMS) was performed on the TOF-SIMS V (ION TOF GmbH) with 30 keV Bi_n_^+^ as the primary ion source. The negative spectra were obtained from 500 × 500 μm^2^ areas by focusing the Bi^+^ primary ions (less than 0.01 pA of pulsed current) in the “burst alignment” mode at a 10 kHz pulsing rate and 120–130 ns pulse width. The surface potential was measured by a Kelvin probe force microscopy with amplitude modulation (KPFM-AM, Multimode 8, Buker) in the tapping mode on a Multi75E-G (budget sensors) probe in air at room temperature.

### Bone marrow-derived mesenchymal stem cells (BMSCs) culture

The femurs obtained from healthy rats were cleaned of adherent muscle and connective tissues. All the experiments were performed following the policy approved by the National Institutes of Health Intramural Animal Use and Care Committee (IACUC-2012-047). The bone marrow cells (BMSCs) were obtained by cutting the ends of the femur and flushing the marrow with 5 mL Dulbecco's modified Eagle's medium (DMEM) containing 5.5 mM glucose (GIBCO, Grand Island, NY, USA), and 10% fetal bovine serum (FBS) (GIBCO) using a syringe fitted with a 25-gauge needle. The cells were seeded in a 75 cm^2^ culture flask (Corning, Lowell, MA, USA) and cultured in a humidified atmosphere (95% air, 5% CO_2_) at 37°C in DMEM containing 10% FBS, 100 U/mL penicillin, and 100 μg/mL streptomycin (GIBCO). The cells were refreshed every 2 days and maintained in the primary culture for 5 to 6 days. When the cells reached confluence, they were trypsinized and placed on new culture plates. To obtain homogeneous populations of BMSCs, single-cell-derived colony cultures were obtained using the limiting dilution technique. Identification of the obtained BMSCs was done and the results are shown in Supplementary Fig. S3.

### Cell staining and observation by microscopy

The BMSCs were seeded on the samples (5 × 10^3^ cells/well) and cultured in the basic medium (DMEM supplemented with 10% FBS) for 3 days. Afterwards, the cells were fixed in 4% paraformaldehyde for 30 minutes, incubated with Phalloidin-TRITC (10 mg/mL, sigma) for 2 hours, and counter-stained with Hoechst 33342 (5 mg/mL, Sigma) to identify the nuclei. Image collection and superimposition were processed by LSM 700 (zeiss, German) and Axio Observer. The experiments were repeated at least three times.

### Flow Cytometry Analysis of the cell cycle and apoptosis

The BMSCs were cultured on tissue culture plastic (noted as Blank), PE, PAr and PArN. Four groups of BMSCs were harvested at day 1 and day 3 and the cells were treated with or without 5 μM 7-xylosyl-10-deacetylpaclitaxel. After incubation for 24 h, the cells were collected and stained with 50 μg/mL DAPI (Partec, Munster, Germany). The cell cycle distribution of 1 × 10^5^ cells was determined by flow cytometry (Partec) and the cell cycle analysis was performed using FloMax software. The fractions of cells in the G0/G1, G2, and S phases of the cell cycle and proliferation index (PI) were analyzed.

Apoptosis was measured by Annexin V-FITC/PI kit (Partec) according to the manufacturer's instructions. After incubation with or without 5 μM 7-xylosyl-10-deacetylpaclitaxel for 48 h, the cells were spun at 1,200 g for 5 min and the supernatant was decanted. The cell pellet was re-suspended with 100 μL of Annexin-V binding buffer and 5 μL of Annexin-V dye and left in darkness at room temperature for 15 min. Following incubation, additional 400 μL of Annexin-V binding buffer were added to each sample. Ten thousand cells were acquired and analyzed by flow cytometry and FloMax software (Partec).

### TOF-SIMS analysis of calcification

After the BMSCs were cultured on Blank, PE, Par, and PArN for 7 days, the samples were rinsed with PBS twice, fixed in 4.0% paraformaldehyde overnight, and washed again with PBS twice. They were dehydrated in a series of ethanol and tert-butanol and freeze-dried for 24 h. SIMS was conducted on a time-of-flight secondary ion mass spectrometer (TOF-SIMS V from ION-TOF GmbH, Munster, Germany) and a Bi_1_^+^ liquid metal ion gun at 30 keV and 45° incident angle was used. The analysis was performed in an area of 200 × 200 μm^2^ corresponding to 256 × 256 pixels. Charge compensation with an electron flood gun was implemented during the analysis and the positive ion spectra were calibrated by the C^+^, CH^+^, C_2_^+^, and C_2_H_3_^+^ peaks.

### Quantitative real time-polymerase chain reaction (PCR) analysis

The total RNA was isolated from BMSCs incubated for 3 days using the Trizol reagent (Invitrogen). Approximately 2–5 μg of total RNA were converted to cDNA by using the Super Script First Strand Synthesis kit (Invitrogen). The real time-polymerase chain reaction (PCR) reactions were performed using the QuantiTect SYBR Green PCR kit (Toyobo, Osaka, Japan) and Applied Biosystems 7500 Real-time PCR Detection System. Three independent experiments were performed for each reaction in triplicate. The primers are shown in Table 1.

### Western Blot analysis

The whole cell lysates were extracted with the lysis buffer (Raybiotech) for western blotting and the protein content of the lysate was determined using a protein assay kit (Beyotime) following the manufacturer's recommended protocol. The proteins were loaded on 10% SDS polyacrylamide gels, transferred to PVDF membranes (Millipore, Billerica, MA), and blocked with 5% nonfat milk powder in PBST (phosphate-buffered saline with 0.1% Tween). The membranes were probed overnight with the following monoclonal primary antibodies: anti-Runx2 (Abcam, Cambridge, UK, 3 μg/mL) and OCN (Abcam, 1:800, Cambridge, UK), and monoclonal antibodies against β-actin from (Zhongshan Jinqiao, China, 1:1000). The membranes were incubated with the anti-mouse horseradish peroxidase-conjugated secondary antibody (Boster, Wuhan, China, 1:1000). The blots were visualized using an enhanced chemiluminescence kit (Amersham Biosciences, Piscataway, NJ) according to the manufacturer's instructions. Densitometry of Western blots was analyzed with Quantity One software and normalized to the respective loading control signal on each blot.

### NOS inhibitor treatment

The cells were seeded at a density of 5,000 cells/cm^2^ in T25 culture flasks, maintained and expanded in DMEM (10% FBS), and allowed to adhere overnight. L-NAME, Sper and L-Can were purchased from Biyuntian (Biyuntian, China). The BMSCs were treated with the eNOS inhibitor (L-NAME, 50 μM); nNOS inhibitor (Sper, 0.5 mM); iNOS inhibitor (L-Can, 1 mM). On day 3, the cells were harvested and subjected to assays for *in vitro* osteogenic differentiation.

### Small interfering RNA (siRNA) transfection

In the siRNA inhibition study, the BMSCs were grown to 60% confluence followed by serum starvation for 12 h. The cells were transfected with siRNA-iNOS(GCAGGACAGCACAGGAAAT) with the GFP gene or scrambled control siRNA oligos (Santa Cruz, USA) at a final concentration of 200 nM according to the manufacturer's instructions. After transfection, the cells were harvested at 72 h for RNA extraction.

### Statistical analyses

The SPSS version 18.0 (Chicago, IL, USA) software was used in the statistical analysis. The data were expressed as means ± SD and p < 0.05 was considered statistically significant. Tukey was used to determine differences between groups.

## Author Contributions

W.Z. and N.L. made equal contributions in this study. W.Z. conceived the experimental plan, performed material preparation and all data analysis, and co-wrote the manuscript. N.L. performed the animal experiments, identification of cell characterizations and biomedical analysis, and wrote the draft manuscript. H.G.S. and B.Z. performed molecular experiments and the analysis. J.L., L.X.S. and H.Y.W. performed materials characterization. J.H.J. and P.K.C. conceived the experimental plan and finalized the manuscript. All authors reviewed and approved the final manuscript.

## Supplementary Material

Supplementary Information Suplementary information

## Figures and Tables

**Table 1:** Primer Sequences for real time PCR

Gene	Primers (F - forward, R - reverse)
ALP	F: 5′-ACAGTGACAGCTGCCCGCAT-3′
	R: 5′-TTGCATCGCGTGCGCTCAGT-3′
BSP	F: 5′-AGACCATGCAGAGAGCGAG-3′
	R: 5′-ACGTCTGCTTGTGTGCTGG-3′
RUNX-2	F: 5′-AGGGCGCATTCCTCATCCCAGT-3′
	R: 5′-AAGACAGCGGCGTGGTGGAA-3′
OCN	F: 5′-TGGCACCACCGTTTAGGGCA-3′
	R: 5′-TTTGGAGCAGCTGTGCCGTC-3′
nNOS	F: 5′-TGAGGTTCTCAGTGTTCGGC-3′
	R: 5′- ATCCTCTCCCCTCCCAGTTC-3′
eNOS	F: 5′- CAAAAGGCACAGGCATCACC-3′
	R: 5′- AAGGCCTCATGCTCTAGGGA-3′
iNOS	F: 5′- ACGGAAGAGACGCACAGGCA-3′
	R: 5′- AAGGCAGCAGGCACACGCAA-3′
TNF-α	F: 5′- ACCTGGCCTCTCTACCTTGT-3′
	R: 5′- GACCCGTAGGGCGATTACAG-3′
GAPDH	F: 5′- GGCACAGTCAAGGCTGAGAATG-3′
	R: 5′- ATGGTGGTGAAGACGCCAGTA-3
