# Supplementary material for: Upregulation of BMSCs Osteogenesis by Positively-Charged Tertiary Amines on Polymeric Implants via Charge/iNOS Signaling Pathway
Source: Sci Rep. 2015 Mar 20;5:9369. doi: 10.1038/srep09369 (PMC4366815; doi:10.1038/srep09369)
Supplement: Supplementary Information — Suplementary information [file srep09369-s1.pdf]

Upregulation of BMSCs Osteogenesis by Positively-Charged Tertiary Amines on Polymeric Implants *via* Charge/iNOS Signaling Pathway

Wei Zhang[1], Na Liu[2], Haigang Shi[1], Jun Liu[1], Lianxin Shi[1], Bo Zhang[2], Huaiyu Wang[3], Junhui Ji[1] & Paul K Chu[3]

[1]Technical Institute of Physics and Chemistry, Chinese Academy of Sciences, Beijing 100190, China,

[2]Stomatology Department of the General Hospital of Chinese PLA, 28 FuXing Road, Beijing 100853, China,

[3]Department of Physics & Materials Science, City University of Hong Kong, Tat Chee Avenue, Kowloon, Hong Kong, China

Correspondence and requests for materials should be addressed to Dr. Wei Zhang (weizhang@mail.ipc.ac.cn), Prof. Junhui Ji (jhji@mail.ipc.ac.cn) and Prof. Paul K Chu (paul.chu@cityu.edu.hk)

Supplementary Data

1. Physical properties of the charged surface with tertiary amines

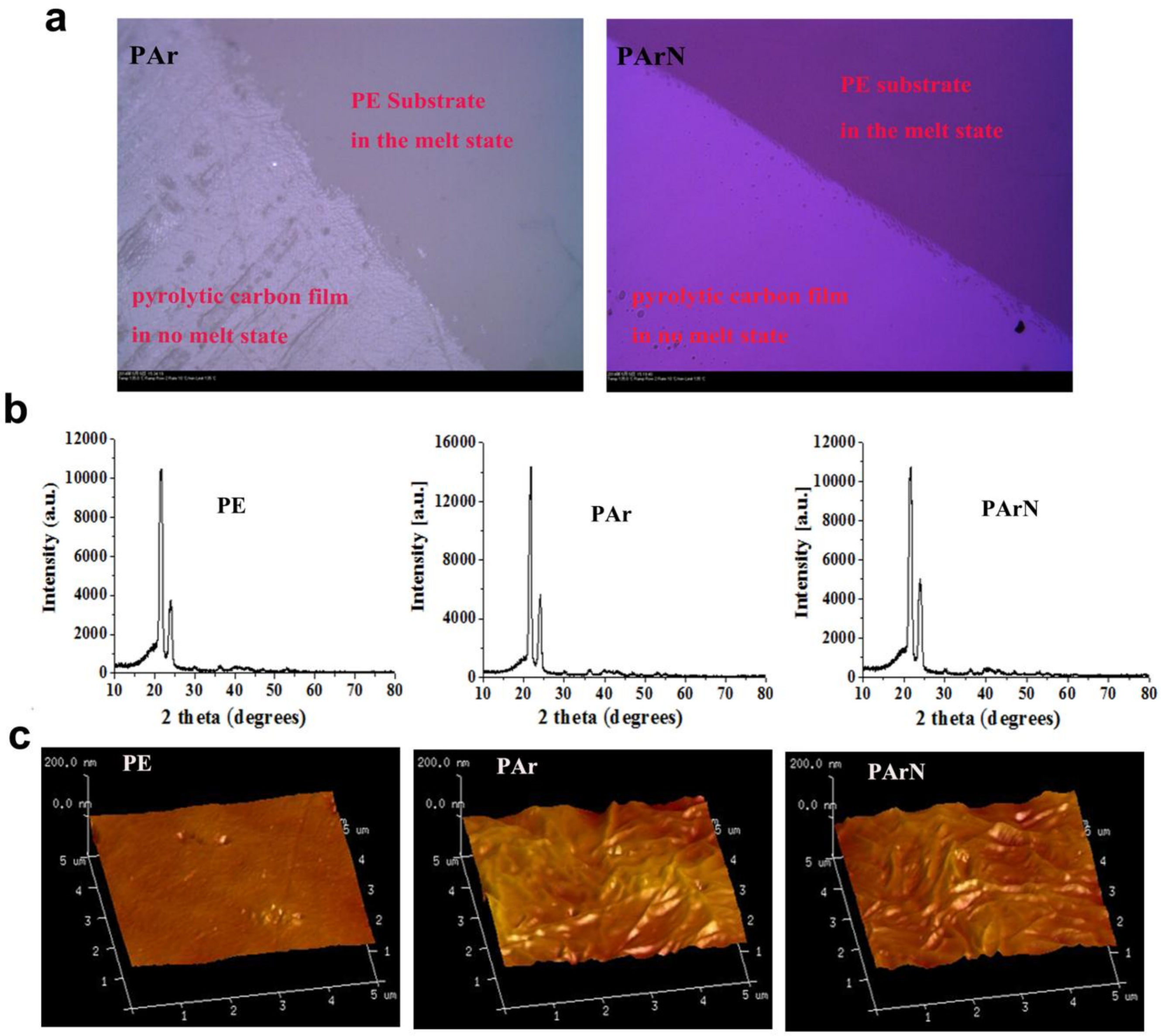

Figure S1 | Physical analysis of PE, PAr, and PArN showing that some crystal structure and large surface roughness are observed from PAr and PArN. (a) Microscopic images of PAr and PArN at 135 ºC magnified 40 times; (b) GIAXRD spectra at an take-off angle of 1º; and (c) AFM images with the RMS surface roughness as follows: PE = 5.7 nm, PAr = 16.1 nm, and PArN = 11.2 nm.

After PAr and PArN were hot-pressed at 140 °C, they were placed on the microscope stage with a heater (Olympus BX51). The pyrolytic carbon films on PAr and PArN did not melt together with the substrate when the temperature rose to 135 °C (Fig. S1a). The grazing-incidence angle X-ray diffraction (GIAXRD, D/max2550HB+/PC) (Cu Kα, 1.5406 nm) was operated at 40 kV voltage and 200 mA and the scanning rate of the X-ray detector was 1 deg/min. The X-ray beam incident direction onto the surface of the samples was perpendicular to the scanning direction of the plasma beam. No crystal phase was observed from the GIAXRD patterns (Fig. S1b). The pyrolytic carbon films on PAr and PArN had a network structure. The surface morphology was determined by atomic force microscopy (AFM, Multimode 8, Bruker) in the tapping mode on a RTESPA (Bruker) probe in air at room temperature. The atomic force microscopy (AFM) images (Fig. S1c) show that the surface on PAr and PArN is rougher because Ar PIII produced cross-linked structures and during cooling, structural reorganization and contraction of the PE substrate under the pyrolytic carbon produced interior stress between the substrate and film making the pyrolytic carbon films wrinkled.

2. Influence of iNOS inhibitor on expressions of three NOS isoforms in BMSCs

When the cell culture medium was added with the iNOS inhibitor (L-Can, 1 mM), it was observed that the BMSCs on blank and PArN had higher expression of eNOS and nNOS, relative to BMSCs without the inhibitor.

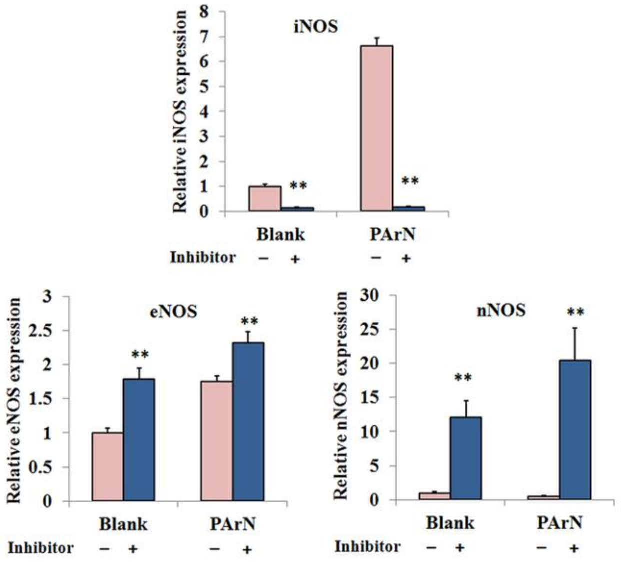

Figure S2 | Expression level of iNOS, eNOS, nNOS in BMSCs co-culutured with iNOS inhibitor. Expressions of eNOS/nNOS/iNOS genes of BMSCs cultured on blank and PArN are measured by real time PCR relative to GAPDH expression and normalized to the expressions on Blank. (**) denote the statistical significance ($p < 0.01$) compared to blank, respectively.

3. Bone marrow mesenchymal stem cells (BMSCs) identification

To identify the mesenchymal stem cells phenotype, fluorescence-activated cell sorting (FACS) analysis was used in the analysis of STRO-1, CD146, CD29, CD90, CD105, CD31, CD34 and CD45 (R&D Systems, Inc.). Induction of osteoblasts and adipocytes was performed as previously described[1]. In brief, the BMSCs were put on a 6-well dish and cultured (1×10^4 cells /well) in DMEM for 1 day. The cells were incubated with adipogenic (0.5 mM methylisobutylxanthine, 0.5 mM hydrocortisone and 60 mM indomethacin; Sigma) or osteogenic medium (100 nM dexamethasone, 50 μg/mL ascorbic acid and 5 mM β-glycerophosphate; Sigma) for additional 14 or 21 days. The medium was replaced every 3 to 4 days. The cells were then fixed with 75% ethanol and stained with Oil Red O solution or 2% alizarin red (Sigma). The cells used in the experiments were passages 2 to 4.

The isolated cells retained a typical fibroblastic spindle shape and expressed the stem cell markers CD29, CD90 and CD105, but were negative for the hematopoietic marker CD31, CD34 and leukocyte marker CD45. The BMSCs were moderately positive for Stro-1 and CD146 (Fig. S3a). These findings are consistent with the phenotypic characteristics of BMSCs populations described previously[2]. The differentiation potential of BMSCs was also investigated by osteogenic and adipogenic induction, followed by alizarin red and Oil Red O staining. Alizarin red staining revealed that BMSCs had formed mineralized nodules (Fig. S3b). Oil red O staining of BMSCs showed that these cells had formed lipid droplets (Fig. S3c). The results demonstrate the potential of BMSCs differentiation into osteoblasts and adipocyte lineages.

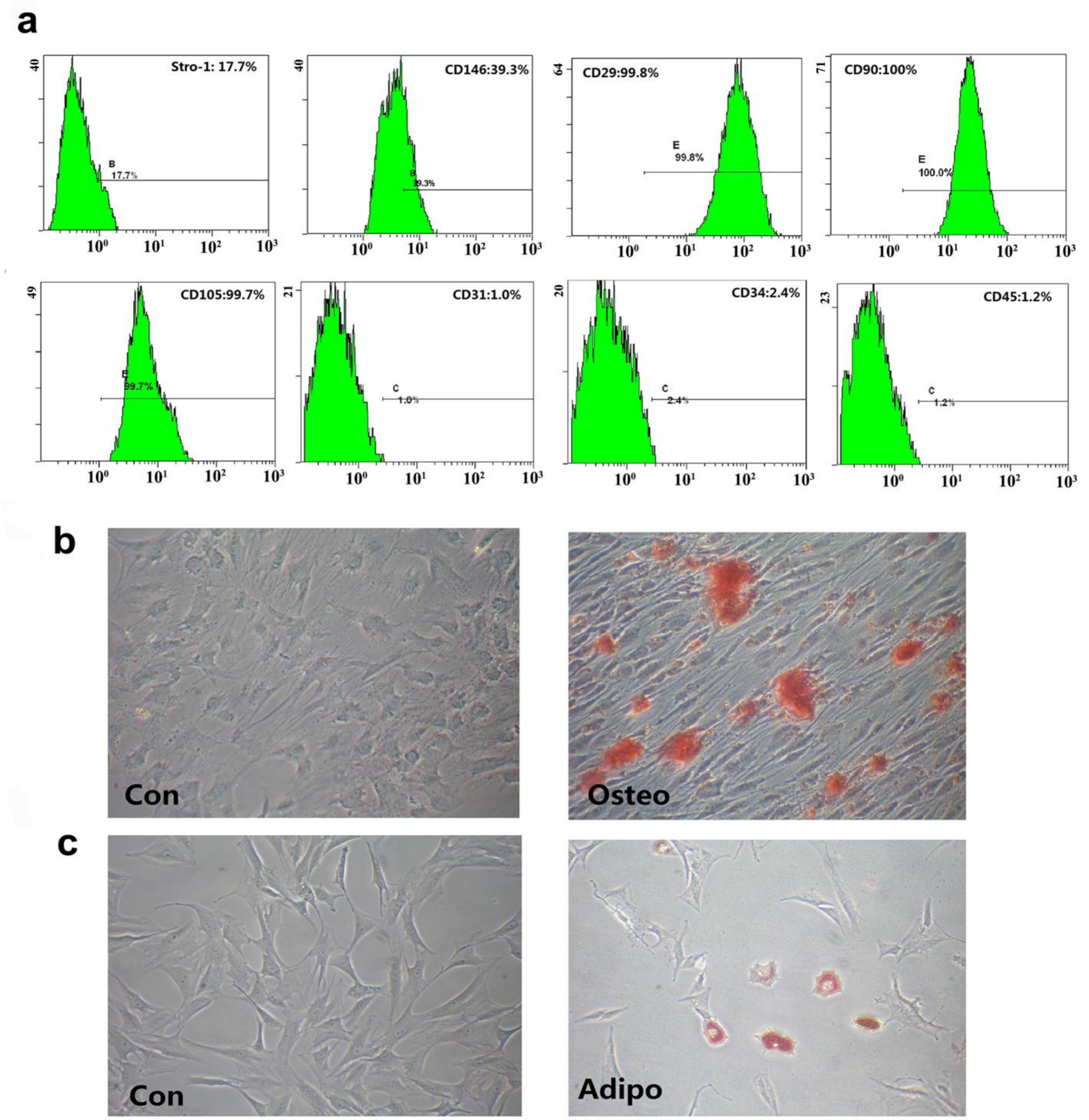

Figure S3 | Identification and differentiation capacity of BMSCs: (a) Flow cytometric analysis of *in vitro* expanded BMSCs reveals expression of STRO-1 (17.7%), CD146 (39.3%), CD29 (99.8%), CD90 (100.0%), CD105 (99.7%), CD31 (1.0%), CD34 (2.4%) and CD45 (1.2%). (b) To investigate the multiple differentiation potential of BMSCs, the cells at passage three were grown in the differentiation medium to induce differentiation *in vitro*. When they were cultured under osteoblastic conditions containing L-ascorbate-2-phosphate,

dexamethasone, and inorganic phosphate for 21 days, mineralized nodules were stained by Alizarin Red. (c) The cells could form lipid clusters which stained positive for Oil Red O following 14 days of adipogenic induction in the presence of 0.5 mM isobutylmethylxanthine, 0.5 μM hydrocortisone, 60 mM indomethacin and 10 mg/mL insulin.

References

1. Seo, B. M. *et al*. Investigation of multipotent postnatal stem cells from human periodontal ligament. *Lancet*. **364**, 149-155, (2004).
2. Tsai, C. L., Wu, P. C., Fini, M. E. & Shi, S. Identification of multipotent stem/progenitor cells in murine sclera. *Invest. Ophthalmol. Vis. Sci.* **52**, 5481-5487, (2011).
